# A single bout of physical exercise improves 1-hour post-load plasma glucose in healthy young adults

**DOI:** 10.1007/s40618-024-02438-8

**Published:** 2024-09-30

**Authors:** Simona Moffa, Gian Pio Sorice, Gianfranco Di Giuseppe, Francesca Cinti, Gea Ciccarelli, Laura Soldovieri, Michela Brunetti, Rebecca Sonnino, Enrico C. Nista, Antonio Gasbarrini, Alfredo Pontecorvi, Teresa Mezza, Andrea Giaccari

**Affiliations:** 1 Centro per le Malattie Endocrine e Metaboliche, Fondazione Policlinico Universitario Agostino Gemelli IRCCS, Roma, Italy; 2 Sezione di Medicina Interna, Endocrinologia, Andrologia e Malattie Metaboliche, Dipartimento di Medicina di Precisione e Rigenerativa e Area Jonica – (DiMePre-J), Università Degli Studi di Bari “Aldo Moro”, Bari, Italy; 3 Dipartimento di Medicina e Chirurgia Traslazionale, Università Cattolica del Sacro Cuore, Roma, Italy; 4 Pancreas Unit, Medicina Interna e Gastroenterologia, CEMAD Centro Malattie dell’Apparato Digerente, Fondazione Policlinico Universitario Gemelli IRCCS, Roma, Italy

**Keywords:** Exercise, Single-bout, 1-h post glucose load, Insulin sensitivity, METs, Metabolic holter

## Abstract

**Purpose:**

Physical exercise is a key component in the treatment of type 2 diabetes and plays an important role in maintaining a healthy glucose metabolism even in healthy subjects. To date, no studies have investigated the effect of a single bout of aerobic physical exercise on glucose metabolism in young, moderately active, healthy adults.

**Methods:**

We performed an OGTT 7 days before and 24 h after a single bout of physical exercise, to evaluate 1-hour post-load plasma glucose and surrogate indexes of insulin sensitivity and insulin secretion.

**Results:**

Glucose levels were significantly reduced after exercise at baseline and one hour after glucose load; similarly, insulin was significantly lower 1 h after glucose load. We found a significant increase in the Matsuda index, confirmed by OGIS index, QUICKI index, and by significant reduction in HOMA-IR. Conversely, we observed a trend to increase in HOMA-B.

**Conclusion:**

This is the first study to evaluate the effect of a single bout of exercise on 1-hour glucose levels following OGTT. We found a significant reduction in 1-hour glucose levels following OGTT together with an increased insulin sensitivity. A single 30-minute bout of aerobic exercise also seemed to improve the insulin secretion pattern. Modifications in beta cell secretory capacity during exercise are likely secondary to an improvement in insulin action in insulin dependent tissues.

**Supplementary Information:**

The online version contains supplementary material available at 10.1007/s40618-024-02438-8.

## Introduction

Physical exercise is now universally acknowledged as a cornerstone for maintenance of good health and metabolic function in both adults and children. It is also a widely recognized therapeutic approach for the management of various chronic diseases and endorsed with particular emphasis in the case of type 2 diabetes, as indicated by the recent American Diabetes Association guidelines [1].

Physical activity and exercise are, however, defined somewhat differently.

Physical activity refers to bodily movement produced by the contraction of skeletal muscle that produces an energy expenditure greater than resting. It can include a broad range of occupational, leisure, and daily activities [2]. Exercise, on the other hand, refers to “planned, structured, and repetitive” physical activity performed to improve or maintain one or more components of physical fitness, [3] The terms physical activity and exercise will be used interchangeably in this paper as we refer to a single session of outdoor aerobic exercise in untrained subjects.

It is well known that exercise influences insulin sensitivity by inducing greater glucose uptake. Specifically, when muscles are active, plasma catecholamines increase rapidly and mobilization of glucose exceeds peripheral glucose uptake. This indicates that during intense exercise other mechanisms are involved in hormonal and substrate mobilization to maintain euglycemia [4].

A substantial body of research has demonstrated the efficacy of physical exercise in ameliorating the metabolic complications associated with obesity by reducing insulin resistance, improving metabolic control, and enhancing the effectiveness of oral hypoglycemic agents. These benefits are especially pronounced with regular and sustained physical activity [1]. One of the primary effects of physical exercise is a reduction in insulin resistance, as exercise potentiates most of the insulin-mediated post-receptor events leading to GLUT-4 translocation to the cell surface. Moreover, the metabolic modifications induced by physical exercise may lead to the reduction of abdominal fat even in children and young adults without diabetes [5].

Physical exercise is, also in healthy subjects, a tool for maintaining glucose metabolism within the normal range. In fact, Myers et al. [6], have shown, in both diabetic patients and healthy subjects, that regular physical activity improves peripheral insulin sensitivity and consequently insulin secretion.

While the role of constant exercise on carbohydrate metabolism is clearly established, the potential effects on insulin secretion and sensitivity are still debated. Previous studies have found either increased or unmodified insulin secretion; although these conflicting conclusions were mostly influenced by the type of population studied. However, less is known about the potential effect of a single bout of exercise.

Several recent studies have focused on the metabolic effects of a single exercise bout in different settings. Heath et al. [7] have demonstrated that most of the beneficial effects of exercise on glucose tolerance and insulin sensitivity in well trained subjects derive from the last bout of exercise (after 11 days without exercise). Newsom and colleagues have shown that in obese adults (BMI 37 ± 1 kg/m2), low intensity exercise improved insulin sensitivity (measured by a hyperinsulinemic clamp) the day after the exercise [8]. Very high-intensity exercise (a single extended sprint) in overweight/obese sedentary men [9] can lead to improved insulin sensitivity (calculated by 75 g OGTT). Endurance interval exercise increases insulin sensitivity (measured with a 75 g OGTT) also in healthy subjects [10]. The discrepancy in the results may be due to the differences among studies in the clinical or fitness status of participants, in the duration or intensity of the chosen exercise patterns, or even the timing of the post-exercise measurements. Further, none of the above has shown results in terms of insulin secretion.

Recently, Shambrook et al. [11] evaluated the difference between accumulated or single bout of walking exercise on acute glycemic response in apparently healthy adults. They found that ten minutes of moderate intensity walking completed 30 min after each meal lowered postprandial dinner glucose concentrations in comparison to no exercise, and reduced glucose by a similar magnitude as a single 30 min bout after the evening meal.

Another recent study explored the impact of a single bout of exercise on insulin-stimulated responses in conduit arteries and capillaries in obese adults. It showed that a single bout of aerobic exercise increased insulin-stimulated pre-occlusion brachial diameter and microvascular blood flow. In addition, acute exercise increased metabolic insulin sensitivity and non-oxidative glucose disposal [12]. Unexpectedly, some authors have demonstrated that the insulin-sensitizing effect of a single bout of exercise in human skeletal muscle is reduced in trained subjects when compared to untrained ones [13]. Thus, it has been known for decades that a single exercise bout can lead to a subsequent increase in insulin sensitivity for glucose disposal and that this effect is evident a few hours after exercise cessation, and can persist for up to 48 h. [14]

To date, however, no studies have explored the effect of a single exercise bout on 1-hour post-load plasma glucose.

Recently, several studies have identified 1-hour post-load glucose concentration during an oral glucose tolerance test as a specific and early predictor of diabetes [15]. In 2007, Abdul-Ghani et al. [16] found that plasma glucose concentration at 1 h during the oral glucose tolerance test is a strong predictor of future risk for type 2 diabetes and that the plasma glucose cutoff point of 155 mg/dl can be used to stratify nondiabetic subjects. In essence, higher values of 1 h post load plasma glucose may identify an intermediate condition between Normal Glucose Tolerance (NGT) and Impaired Glucose Tolerance (IGT) characterized by worse insulin sensitivity, reduced beta-cell glucose sensitivity, and reduced beta-cell rate sensitivity. [17]

Hence our idea to evaluate the effect of a single exercise bout (SEB) of aerobic physical exercise specifically on 1-hour post-load plasma glucose through an oral glucose tolerance test (OGTT) administered the day after the exercise session, in young, healthy, moderately active adults. We hypothesized that acute aerobic exercise would reduce 1-hour post-load plasma glucose, improve insulin sensitivity, and consequently beta-cell secretive function. Thus, since 1-hour post-load plasma glucose is a strong predictor of diabetes risk, this could be further confirmation of the effectiveness of even occasional exercise in reducing the risk of developing type 2 diabetes mellitus.

Therefore, the primary aim of our study was to investigate the acute and direct effect of a single bout of aerobic exercise especially on 1-hour post-load glucose during Oral Glucose Tolerance Test in young, healthy, normal weight volunteers with a sedentary/moderately active lifestyle (not practicing competitive sports, just recreationally active).

## Research design and methods

A total of 14 women and 18 men volunteered to participate in this study and were recruited. All participants were aged between 20 and 35 years, were normal weight or marginally overweight (BMI 18–28 kg/m2), healthy, with no sign of diabetes and none were on medications. None of the participants could be considered trained, as they were all sedentary or mildly active only during leisure time. These participants were chosen for the study since they represented a population that could be investigated after a single bout of physical exercise, as the obtained results would not be influenced by prior training or structured repetitive physical activity.

The study protocol was approved by the local ethics committee (protocol number 8497/15), and all participants provided written informed consent, which was followed by a comprehensive medical evaluation.

Exclusion criteria included fasting blood glucose concentration ≥ 100 mg/dl (5.6 mmol/L), pregnancy or nursing, altered renal or hepatic function, any history of cardiovascular, neurological, hematological, endocrine, or pulmonary diseases, any competitive sport practice and positive beta-cell autoimmunity. Those who were unable to understand and sign the informed consent were also excluded.

Participants were asked not to undertake any exercise outside of the lab sessions and to maintain their normal lifestyle for the entire duration of the study. Each participant underwent an initial evaluation to collect medical history and measurements, namely height, body mass, waist, and hip circumference. All participants were interviewed for demographic information, personal medical history (i.e., diseases and medications), and lifestyle habits (smoking, physical activity, and alcohol use). Furthermore, using the Meta Dieta nutrition software, we estimated the nutritional status of the enrolled subjects so as to be able to prescribe a controlled diet for each participant on the day before the second oral glucose tolerance test.

On the same day, all subjects were administered a first baseline oral glucose tolerance test after at least 4 days of physical inactivity.

Participants reported at our outpatient clinic at 08:00 a.m. following a 12-hour overnight fast. Blood tests were run to evaluate liver (aspartate aminotransferase and alanine aminotransferase) and kidney (creatinine) function, complete blood count, lipid profile (total cholesterol, high-density lipoprotein, and triglycerides), vitamin D and thyroid function (results are shown in Table 1). Participants were then administered a standard 75 g oral glucose tolerance test after a 12 h overnight fast, with measurement of glucose and insulin at 0, 30, 60, 90, and 120 min after the glucose load. We determined insulin levels using a commercial RIA kit (Medical System, Immulite DPC, Los Angeles, CA). Plasma glucose concentrations were determined by the glucose oxidase technique, using a glucose analyzer (Beckman Instruments, Palo Alto, CA, USA). All samples were analyzed at the central laboratory of the Fondazione Policlinico Universitario Agostino Gemelli IRCCS Hospital.

Seven days after baseline assessment, all participants were required to perform a single bout of physical exercise. Following the recommendations of the American Diabetes Association [18], we adopted 30 min as the standard exercise time. The 30-minute aerobic activity session was supervised by the investigators (SM and GPS) and consisted of a light jog for all participants.

Each performance was monitored using a metabolic holter (SenseWear^®^ Armband, Body Monitoring System), a validated instrument that we used to quantify the following exercise parameters: Total Energy Expenditure [19], Active Energy Expenditure and METs. The MET concept (Metabolic Equivalent) is a simple and practical procedure for expressing energy expenditure during physical activity as a multiple of the resting metabolic rate. Thus, one MET corresponds to the oxygen consumed while sitting at rest and is equal to 3.5 ml O2 per kg body weight/min. Exercise intensity was adjusted to require between 60 and 65% of V ˙ O2max by monitoring heart rate during the exercise sessions. This was accomplished by using the heart rate values obtained at 60–65% of the measured VO2 max.

On the day in which they performed the single bout of physical exercise, i.e. before the second oral glucose tolerance test, to ensure reliable glycemic levels for the glucose tolerance evaluation, participants followed a controlled balanced diet consisting approximately of 50% carbohydrates, 25% lipids and 25% proteins. (details in Supplementary materials)

The morning after the exercise session, subjects were administered a second oral glucose tolerance test, following the same procedure as the first. Blood glucose and insulin values after the single exercise bout were compared with corresponding values obtained during the period in which the same subjects had not engaged in any type of physical activity in the previous 4 days. Thus, each participant acted as his/her own control.

For the present study, insulin sensitivity and secretion were estimated using surrogate indices derived from oral glucose tolerance test glucose and insulin assays. Whole body insulin sensitivity was estimated with the Matsuda index and the De Fronzo composite whole body insulin sensitivity index (ISI-COMP) [20]. This calculation uses fasting plasma glucose (mg/dl), plasma insulin (µU/l) and the average plasma glucose and insulin values over the 30, 60, 90 and 120 min from an OGTT. This index is highly correlated (*r* = 0.89; *p* < 0.005) with the insulin sensitivity measures derived from the hyperinsulinemic euglycemic clamp. Moreover, we estimated the Oral Glucose Insulin Sensitivity index (OGIS), an index calculated using a model-derived formula from oral glucose tolerance test glucose and insulin concentrations based on a physiological insulin-glucose model. The OGIS is comparable to the glucose clearance calculated during a clamp and has been validated against the clamp method in lean, obese, IGT and diabetic subjects [21]. For post-load evaluation, we also calculated the total area under the curve (AUC) using the trapezoidal method.

Additionally, the Homeostasis Model Assessment estimated Insulin Resistance (HOMA - IR) was calculated using fasting insulin (µU/ml) x fasting glucose (mg/dl1)/405, which also has a strong correlation with the clamp technique [22]. From fasting glucose and insulin values, we also obtained the quantitative insulin sensitivity index [23] (QUICKI). We used HOMA - B to assess beta cell function ((HOMA - B (20 fasting insulin, pmol/L)/ (fasting glucose, mmol/L − 3.5) [24].

All data are expressed as mean ± SD, unless otherwise indicated. Since samples were normally distributed, differences in means were tested by 2-tailed Student’s t test.

## Results

All study participants underwent general blood tests pre- and post-exercise, and a comparison of the baseline and post-exercise values showed no significant differences. Table 1 illustrates these results together with the general characteristics of the study population (age, sex, BMI).

**Table 1:** Characteristics of study population and general blood test values. Data represents mean ± SD

Parameter	Baseline	After exercise	*P* Value
Age (years)	**35 ± 3**	**-**	**-**
Sex	**14 W/ 18 M**	**-**	**-**
BMI (kg/m^2^)	**22.9 ± 2**	**-**	**-**
Waist circumference (cm)	**75.4 ± 8**	**-**	**-**
FPG (mg/dl)	**82.8 ± 6**	**78.5 ± 8**	**NS**
2 h-PPG (mg/dl)	**87.4 ± 19**	**87.9 ± 18**	**NS**
HbA1c (%/mmol/l)	**34.5 ± 2.4**	**34.8 ± 2.8**	**NS**
AUC glucose (mg/dl x min)	**13,258 ± 2582**	**12,857 ± 1989**	**NS**
AUC insulin (µIU/ml x min)	**5047 ± 2739**	**4579 ± 1860**	**NS**
GOT (IU/L)	**30 ± 6**	**31 ± 5**	**NS**
GPT(IU/L)	**32 ± 4**	**33 ± 6**	**NS**
Creatinine (mg/dl)	**0.94 ± 0.6**	**0.9 3 ± 0.7**	**NS**
Total cholesterol (mg/dl)	**167.6 ± 32**	**162.2 ± 24**	**0.04**
HDL-C (mg/dl)	**61.2 ± 16**	**61.2 ± 15**	**NS**
Triglycerides (mg/dl)	**74 ± 48**	**77.8 ± 45**	**NS**
LDL-C (calculated) (mg/dl)	**91.6 ± 25**	**84.5 ± 22**	**0.01**
Vitamin D (ng/ml)	**18 ± 7**	**14.3 ± 6**	**NS**
TSH (mcUI/ml)	**1.7 ± 0.6**	**1.6 ± 0.8**	**NS**

Table 1: main clinical features of study population (age, sex, BMI, waist circumference), and metabolic parameters before and after exercise: FPG (Fasting Plasma Glucose), 2 h-PPG (Post Prandial Glucose), AUC of glucose and insulin. NS (Not Significant).

**Fig. 1:**
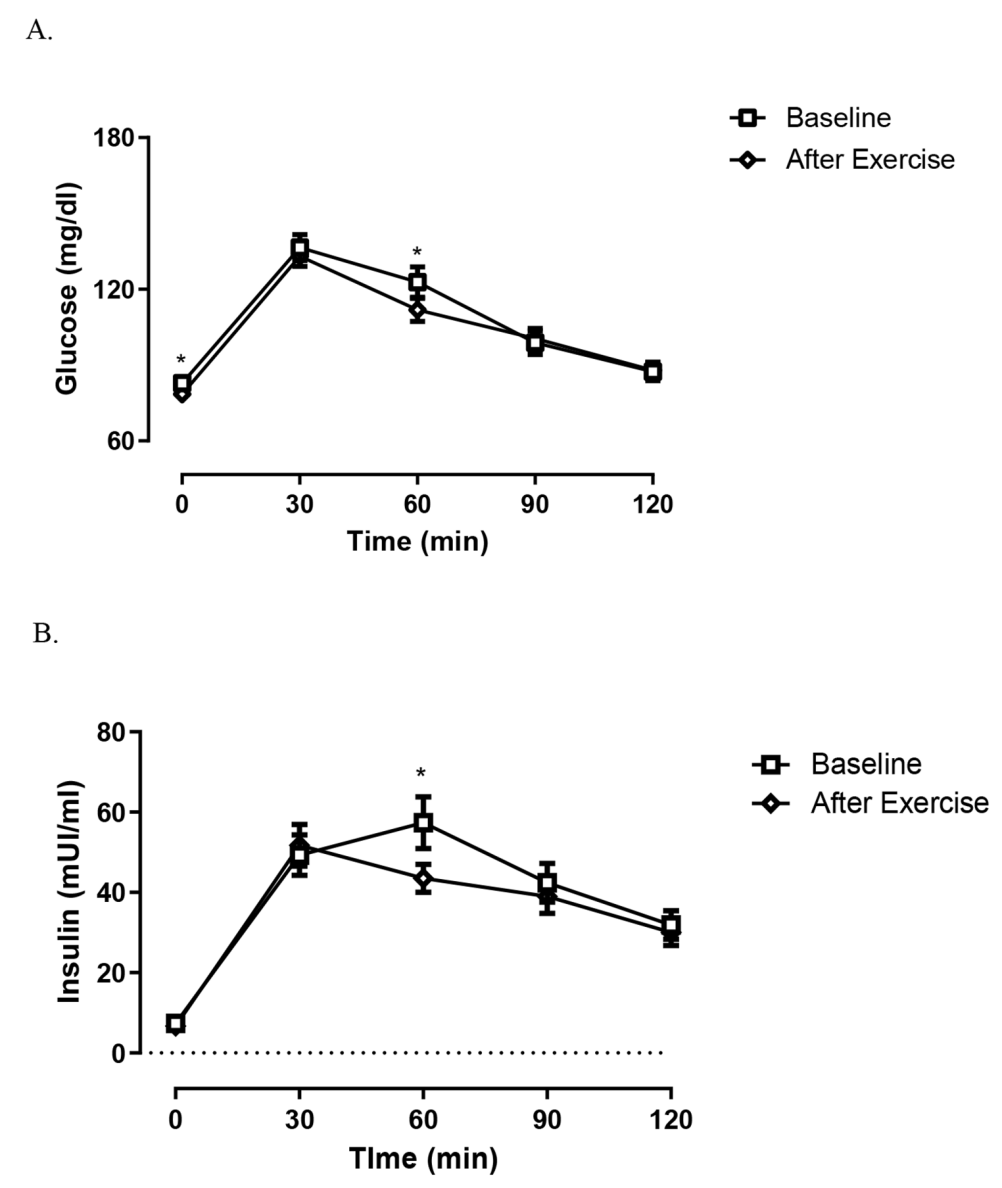
Glucose (**A**) and Insulin (**B**) levels following OGTT. * *p* < 0.05. Data represent means ± SE

All participants successfully completed the protocol. Time series for plasma glucose and plasma insulin concentrations, at baseline (B) and after exercise (AE) during OGTTs are shown in Fig. 1.

Glucose levels were significantly reduced after the single bout of physical exercise at baseline and one hour after glucose load (Basal glucose before 82.8 ± 5 mg/dl vs. after exercise 78.5 ± 7 mg/dl, *P* = 0.002; one hour glucose levels following OGTT before 122.8 ± 34 mg/dl vs. after exercise 111.8 ± 25 mg/dl, *P* = 0.03).

Similarly, insulin was significantly lower one hour after glucose load considering post exercise plasma levels (Before 57.4 ± 37 µUI/ml vs. After Exercise 43.5 ± 19 µUI/ml, *P* = 0.01), while no significant differences were found in the Area Under the Curve of both glucose and insulin between baseline and post exercise evaluation (Table 1).

**Fig. 2:**
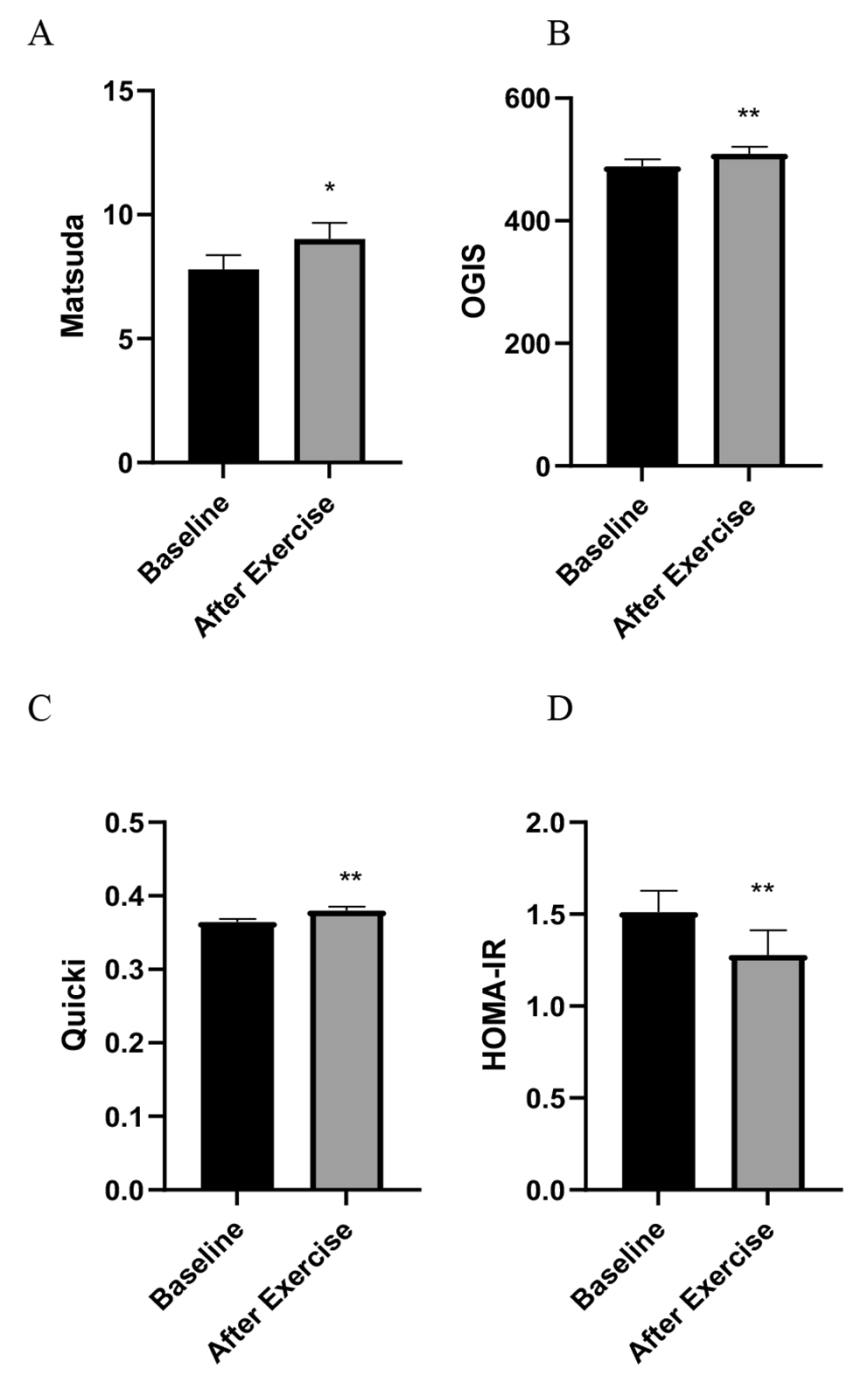
Surrogate indices of insulin sensitivity evaluated during OGTT: Matsuda (**A**), OGIS (**B**) and from baseline values of insulin and glucose: Quicki (**C**); HOMA-IR (**D**). * *p* < 0.05, ** *p* < 0.01. Data represent means ± SE

**Fig. 3:**
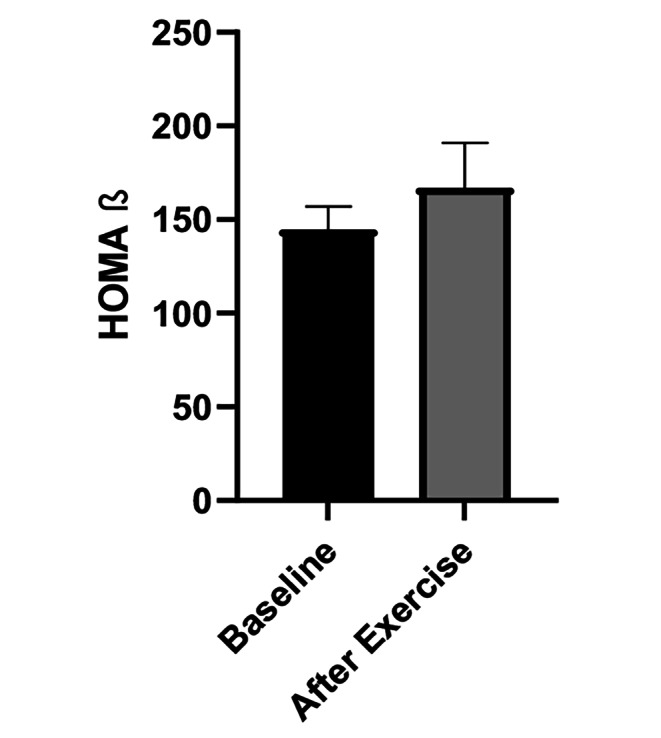
HOMA B. Data represent means ± SE

On the other hand, assessing surrogate indices of insulin sensitivity (Fig. 2), we found a significant improvement in insulin sensitivity after exercise. In particular, there was a significant increase in the Matsuda index (B: 7.79 ± 3.3 vs. AE: 9.02 ± 3, *P* = 0.02), that was also confirmed by the OGIS index (B: 488.71 ± 63.9 vs. AE: 509.36 ± 65.05, *P* = 0.04). The QUICKI index was also significantly higher (B: 0.36 ± 0.02 vs. AE: 0.38 ± 0.03, *P* = 0.001), while we observed a significant reduction in HOMA-IR (B: 1.51 ± 0.66 vs. AE: 1.28 ± 0.76, *P* = 0.04). Conversely, we observed a trend to increase in HOMA-B (Fig. 3), following exercise (B: 144.7 ± 70; AE: 167.1 ± 133, P = NS).

The metabolic holter used during physical exercise showed that enrolled subjects performed a single bout of physical exercise of 32 ± 15 min on average; the average intensity of the physical exercise was 6.9 ± 1.5 METs; total energy expenditure was 365 ± 125 Kcal, while active energy expenditure was 352 ± 106 Kcal, with no significant differences between the two parameters (p = NS), meaning that participants were exercising during the entire time the armband was worn.

We also compared the effect of a single bout of physical exercise in males vs. females in terms of both plasma glucose and surrogate indices of insulin sensitivity and insulin secretion, but found no significative differences.

## Discussion

Several studies, some older, others relatively recent, have scientifically demonstrated the benefits of exercise. Apart from the molecular mechanisms involved, the main, and most widely accepted, effect of exercise on glucose metabolism is represented by the marked enhancement of insulin sensitivity [25, 26], even in diabetic subjects [27, 28].

Most of the studies on exercise and glucose metabolism have focused on the chronic effect of exercise. On one hand, exercise seems to improve the action of insulin [29], while plasma insulin response to a given hyperglycemic stimulus is decreased [30], suggesting an improvement in insulin action. However, few data are available on the acute effect of exercise on glucose metabolism. Furthermore, mainly due to the differences in the metabolic tests used to assess insulin response, the type of exercise performed, and the population studied, in depth knowledge as to how physical exercise impacts insulin production is still lacking.

Our study demonstrated a significant reduction in both fasting and 1-hour glucose levels following OGTT, suggesting a more effective insulin secretion, after a single bout of light aerobic exercise. We also observed that a single 30-minute bout of aerobic exercise in young and healthy, untrained subjects, improved insulin sensitivity with a concomitant improvement in glucose levels and a positive trend in beta cell secretory response [31, 32].

It is rather difficult to accurately distinguish between improved insulin sensitivity and improved insulin responsiveness in vivo. However, some studies have attempted to verify the underlying mechanisms in muscles perfused or incubated in vitro with different insulin concentrations, demonstrating that exercise improves both the sensitivity and responsiveness of skeletal muscle to insulin [33]. The increase in muscle insulin sensitivity after a single bout of exercise occurs as the acute insulin-independent stimulation of glucose transport by the exercise wears off. Animal models have shown that the increase in muscle insulin sensitivity persists as long as muscle glycogen supercompensation does not occur [34].

In a 1985 study, Arciero et al. showed that compared to a short-term low calorie diet (no more than 10 days), short-term exercise is more effective in enhancing insulin action in adult individuals with abnormal glucose tolerance (impaired glucose tolerance or type 2 diabetes) [35].

Another study compared the effects of aerobic and resistance training on several metabolic risk factors, concluding that both types of training improve metabolic features in type 2 diabetic patients in a similar way and that improved glucose control was mainly driven by an enhanced insulin sensitivity, with no significant changes in beta cell function [36].

Conversely, Dela et al. demonstrated that aerobic training in trained subjects with type 2 diabetes may lead to an enhanced beta cell function, even though the effect of training depends on the remaining beta cell secretory capacity, i.e. training improves beta cell insulin secretion only on condition of moderate secretory capacity. In contrast, in diabetics with low secretory capacity, training showed no effects on beta cell function [37].

On the other hand, Dunstan et al. found that short-term circuit training (3 days/week for 8 weeks) in metabolically well-controlled type 2 diabetes subjects, did not improve insulin area under the curve after oral glucose load [38], thus suggesting the presence of other mechanisms behind the modification of beta cell secretory capacity after exercise or, at least, a more effective secretion of insulin (same amount of released insulin, better peripheral effect on insulin-dependent tissues).

Although in daily clinical practice 2-hour post-load glycaemia is used for clinical reasons (diagnosis of alterations of glucose tolerance/metabolism), growing evidence has highlighted the importance of evaluating glycaemia after 1 h. In fact, it has been suggested that higher 1 h glucose level at OGTT is a strong predictor of future risk of type 2 diabetes [16]. Moreover, levels of 1-hour post-load plasma glucose higher than 155 mg/dl have been associated with different types of subclinical organ damage, such as chronic kidney disease and carotid atherosclerosis [39–41], well known independent predictors of increased cardiovascular mortality. Thus, improvement in 1-hour post-load plasma glucose following a single session of aerobic physical activity suggests that exercise could have a direct effect on T2D risk and cardiovascular risk.

To date, few studies have evaluated the effect of a single bout of aerobic physical exercise on glucose and insulin metabolism in healthy subjects. Shambrook et al. [11] demonstrated that short bouts (10 min) of exercise after each meal are better than a single 30-minute bout to minimize post-meal glucose elevation. Similarly, Kao and colleagues [42] examined the effects of a single bout of short-duration, high-intensity exercise and long duration, low intensity exercise on insulin sensitivity and the adiponectin/leptin ratio in individuals with different body mass indices (BMIs) who did not exercise regularly; they found that HOMA - IR improved significantly for both exercise patterns in the normal weight group and for the high intensity pattern in the obese group. However, no other insulin resistance indexes or insulin secretion parameters were analyzed.

Another study investigated the effect of a single bout of aerobic exercise on short-term high fat diet-induced postprandial glucose and incretin metabolism during an oral glucose tolerance test. They found that although exercise did not improve postprandial glucose and insulin metabolism during the OGTT, it did however normalize the increased postprandial GLP-1 levels induced by the high fat diet [43].

Engeroff et al. [44] found that, after a meal mimicking a typical Western breakfast and a sedentary period of 4 h, regular short exercise breaks, but not exercise prior to sitting, could lower blood insulin levels in premenopausal, healthy, female participants. Given these conflicting results, due to the heterogeneity of the population enrolled, duration of exercise and concomitant diet, we decided to enroll healthy, normal weight, untrained volunteers, in order to perform our metabolic assessment.

We metabolically studied all enrolled subjects before and after performing a single bout of aerobic exercise monitored using a metabolic holter. After the single bout of physical activity, we found an improvement in glycemic values, particularly those measured at fasting state and 1 h post-oral glucose load.

Insulinemic values mirrored this trend, given that 1 h post-load levels were also significantly reduced. Moreover, assessing the surrogate indices of insulin sensitivity and insulin secretion, we found that secretion improved after exercise, although this improvement did not reach statistical significance. This seems to be due to increased peripheral sensitivity to insulin, as evidenced by the statistically significant improvement in all the indices of insulin resistance considered [45, 46]. Specifically, we found an improvement in both whole-body insulin sensitivity, assessed by Matsuda index and confirmed by the OGIS index, and in hepatic insulin sensitivity considering HOMA - IR and QUICKI indexes (obtained only from fasting glucose and insulin levels).

Even though we have still not reached a complete understanding of the effect of physical exercise on insulin production, mainly due to the differences in the metabolic tests used to assess insulin response, the type of exercise and the population studied, our study demonstrated the acute improvement of insulin sensitivity after exercise, primarily influenced by the improved capacity of beta cells to produce insulin.

Our study had some limitations: the sample size was limited, and C-peptide values, which would have allowed us to improve our understanding of insulin secretion, were not measured.

Despite these potential limitations, the strengths of our study include the enrollment of young, healthy, untrained subjects, who represent an ideal model in which to verify the pathophysiology of insulin secretion and sensitivity. Furthermore, the use of the armband allowed the precise monitoring of physical activity and the normalization of the results for energy expenditure.

## Conclusions

In conclusion, we found that in young, healthy, normal weight and untrained subjects, a single 30-minute bout of outdoor aerobic exercise significantly reduced both fasting and 1 h glucose levels following 75 g OGTT, suggesting more effective insulin secretion. Moreover, we found an improvement in insulin sensitivity, as demonstrated by the improvement in both whole-body insulin sensitivity surrogate indices (Matsuda index and OGIS index), and in hepatic insulin sensitivity (HOMA-IR and QUICKI indexes). A single bout of 30-minute aerobic exercise also seemed to improve the insulin secretion pattern. The modifications in the secretory capacity of beta cells during exercise are likely secondary to an improvement in insulin action in insulin dependent tissues.

## Electronic supplementary material

Below is the link to the electronic supplementary material.

Supplementary Material 1

Supplementary Material 2

## Data Availability

The datasets generated and/or analyzed during the current study are available from the corresponding author on reasonable request.

## Abbreviations

SEB: Single exercise bout
OGTT: Oral glucose tolerance test
BMI: Body mass index
MET: Metabolic equivalents
HOMA: Homeostasis model assessment
QICKI: Quantitative insulin sensitivity index
B: Baseline
AE: After exercise
AUC: Area under the curve
T2D: Type 2 diabetes
HFD: High fat diet
