# Supplementary material for: A single bout of physical exercise improves 1-hour post-load plasma glucose in healthy young adults
Source: J Endocrinol Invest. 2024 Sep 30;48(2):455–64. doi: 10.1007/s40618-024-02438-8 (PMC11785650; doi:10.1007/s40618-024-02438-8)
Supplement: Supplementary file 1 — Supplementary Material 1 [file 40618_2024_2438_MOESM1_ESM.docx]

**SUPPLEMENTARY MATERIAL**

**A single bout of physical exercise improves 1-hour post-load plasma glucose in healthy young adults**

**Simona Moffa^1^**, Gian Pio Sorice^2^**, Gianfranco Di Giuseppe^1,3^, Francesca Cinti^1,3^, Gea Ciccarelli^1,3^, Laura Soldovieri^1,3^, Michela Brunetti^1,3^, Rebecca Sonnino^1,3^, Enrico C. Nista^3,4^, Antonio Gasbarrini^3,4^, Alfredo Pontecorvi^3^, Teresa Mezza^3,4^, Andrea Giaccari^1,3^**

1. **Centro per le Malattie Endocrine e Metaboliche,** **Fondazione Policlinico Universitario Agostino Gemelli IRCCS, Roma, Italy**
2. **Sezione di Medicina Interna, Endocrinologia, Andrologia e Malattie Metaboliche, Dipartimento di Medicina di Precisione e Rigenerativa e Area Jonica – (DiMePre-J), Università Degli Studi di Bari “Aldo Moro”, Bari, Italy**

**3 Dipartimento di Medicina e Chirurgia Traslazionale, Università Cattolica del Sacro Cuore, Roma, Italy**

**4 Pancreas Unit, CEMAD Centro Malattie dell'Apparato Digerente, Medicina Interna e Gastroenterologia, Fondazione Policlinico Universitario Gemelli IRCCS, Roma, Italy**

*** Correspondence: teresa.mezza@unicatt.it (Teresa Mezza, MD PhD)**

**** Simona Moffa and Gian Pio Sorice contributed equally to this work.**

Table 2. Energy and macronutrient intake of enrolled patients, the day before OGTT evaluation.

| Parameter | Daily intake | % total Kcal |
| --- | --- | --- |
| Energy (kcal) | 1.881 ± 394 |  |
| Carbohydrates (g) | 227 ± 52 | 45± 6 |
| Free sugar (g) | 35 ± 12 | 10 ± 3 |
| Protein (g) | 120 ± 14 | 22 ± 5 |
| Fat (g) | 70 ± 16 | 28 ± 5 |
