# Supplementary material for: A single bout of physical exercise improves 1-hour post-load plasma glucose in healthy young adults
Source: J Endocrinol Invest. 2024 Sep 30;48(2):455–64. doi: 10.1007/s40618-024-02438-8 (PMC11785650; doi:10.1007/s40618-024-02438-8)
Supplement: Supplementary file 2 — Supplementary Material 2 [file 40618_2024_2438_MOESM2_ESM.docx]

Dear Editor,

I enclose a revised version of the manuscript "A single bout of physical exercise improves 1-hour post-load plasma glucose in healthy young people". We would appreciate your opinion as to its suitability for publication in the Journal in its revised form.

We have addressed all the points raised by the reviewers.

I look forward to your comments.

Regards,

Teresa Mezza

Reviewer #1: The manuscript by Simona Moffa et al describes the effect of a single bout of physical exercise on 1 hour post-load plasma glucose in healthy young people. The manuscript deals with a very important scientific and clinical issue, it is well written, results are clearly presented and supported by data. The Discussion is accurate and all relevant bibliography had been quoted.

I have one single suggestion: It would be very interesting to compare the effect of the single bout of physical exercise in males vs females. In fact gender differences were demonstrated between genders mainly related to the type of physical exercise performed.

Thank you for the interesting comment. We compared the effect of a single bout of physical exercise in males vs females on both plasma glucose and surrogate indices of insulin sensitivity and insulin secretion, but we found no significative difference. However, our sample is rather small to observe any stratification by sex, so a larger sample would be needed to evaluate any gender differences. We take this opportunity to confirm that all recruited subjects performed the same type of physical exercise. We reported this at line 224-225.

Reviewer #3: In this study, Simone Moffa et. al investigated the effects of a single bout of physical exercise in 1 hour post-load plasma glucose on a population of young, moderately active healthy adults. The study is interesting, adopts well-conceived methods, and results are convincing. However, some minor revisions are requested to further improve the impact of the manuscript:

* Introduction should be improved. The main issues are:

o In line 105, authors report that "it has been known for decades that a single exercise bout can lead to a subsequent increase in insulin sensitivity… for up to 48 h". However, the authors should clearly specify the absolute novelties of the work. The introduction should focus more on data that are still missing in the literature and that this study will provide for the first time, making it novel and original.

o Soon after, in line 107, authors describe the rationale of the study, reporting that "our idea to confirm the effect of a single exercise bout…". I would suggest avoiding statements such as "to confirm" because might lead to believe that the work reiterates already known notions.

o The rationale of the study described in the previous point is repeated in line 118-122. I suggest revising introduction to be less redundant.

We thank the reviewer for the comments. We revised the Introduction focusing more on data that are still missing, and emphasizing the novelty of our study (line 108-115; 118-121).

We changed the statement "to confirm" in "to evaluate" (line 116).

We also eliminated repetitions in the Introduction to make it easier to read.

* It is not clear why table 2 is separated from table 1 since both contains haematochimic parameters at baseline and after exercise. A single table might be easier to read.

* Units of measurements are missing for some values in table 1 (AST, ALT, vitamin D).

We thank the reviewer for the suggestion. We have now merged the two tables and added the missing data (line 192-197).

* Line 153: "all samples were analyzed at our central laboratory". I would suggest indicating the name of the laboratory since several affiliations from different departments participated to the study.

We thank the reviewer for the observation. We have now specified the name of the hospital where blood samples were analyzed. (line 154-155)

* In the abstract, "conclusion" repeats what has already been said in the results and appear slightly redundant. I would suggest rephrasing by indicating the clinical impact of the study results and novelties of the study.

We thank the reviewer for the suggestion. We have now modified the conclusion in the abstract. (line 48-52).

* In methods, line 125, authors report that participants "were normal weight or minimally overweight (BMI 18-<30 kg/m^2)". Given that overweight range from 25 to 30, authors could indicate the actual range of BMI of recruited patients (should be around 21-25 according to table 2) to highlight that participants were at most "minimally overweight".

We thank the reviewer for the comment. We have modified the text, inserting the correct BMI range (line 127). Of all the subjects enrolled, only 2 had a BMI just over 27, 4 subjects had a BMI over 25, while all the others had a normal BMI. Thus, the group is quite homogeneous, which is why the standard deviation shown in the table is relatively small.

We have now also specified that data in the results session and in Table 1 are represented as mean ± SD, while in the figures data are represented as mean ± SE.

* In methods, it has been reported that the nutritional status of enrolled subjects has been assessed. However, the rationale for this measurement is not clear. It appears that the aim was to ensure reliable glycemic levels for the glucose tolerance test rather than confirm that recruited patients had healthy eating habits, but this should be better explained. If it is possible for authors, to provide average values of nutrient intakes from patients in supplemental materials would add strength to the study.

We thank the reviewer for pointing this out. We explained the reason for the controlled calorie intake the day before OGTT evaluation (line 168-169). We have detailed energy and macronutrient intake in the Supplementary materials.

* In line 189 authors reported that all data were adjusted for the measured energy expenditure. However, it is not clear how this adjustment was made and calculated and should be better described in methods. I would also suggest specifying that we are referring to energy expenditure measured with the metabolic holter. In this regard, I would like to ask the authors if they have found any associations between energy expenditure measured during exercise and the improvements in the glycemic profile/insulin sensitivity.

We thank the reviewer for giving us the opportunity to better clarify some points.

Data shown in the manuscript are not adjusted for the measured energy expenditure, so we removed the confusing sentence.

We attempted to see if there was a correlation between energy expenditure measured during exercise and glucose profile and insulin sensitivity indices, but we did not find any statistically significant difference.

I would also suggest to revise English and some typos (e.g.: line 235, 335 remove hyphens between words).

We revised English and corrected grammar errors.
